# Effects of Qishen Yiqi dropping pills plus conventional Western medicine on cardiac function and quality of life of patients with chronic heart failure

**DOI:** 10.3389/fmed.2026.1769518

**Published:** 2026-04-14

**Authors:** Zhongping Du, Yueyue Jing

**Affiliations:** 1 Department of Internal Medicine II, Shahe Hospital, Beijing, China; 2 Department of Cardiovascular Medicine, Yan’an People’s Hospital, Yan’an, China

**Keywords:** cardiac function, chronic heart failure, exercise endurance, inflammation, Qishen Yiqi dropping pills

## Abstract

**Aim:**

To assess the impact of combining Qishen Yiqi dropping pills (QSYQ) with conventional Western medicine on the cardiac function and quality of life in patients with chronic heart failure (CHF).

**Methods:**

This was a randomized controlled trial (RCT), and 120 patients with CHF were recruited and randomly divided into the control group (receiving conventional Western medicine treatment) and study group (receiving QSYQ and conventional Western medicine treatment) at 1:1 ratio. The primary outcome was clinical efficacy, and the secondary outcomes were cardiac function, exercise tolerance, quality of life, inflammatory markers, and adverse reactions.

**Results:**

The study group demonstrated a higher total effective rate than the control group (91.67% vs. 70%, *p* = 0.002). Also, left ventricular end-systolic diameter (LVESD) and left ventricular end-diastolic diameter (LVEDD) were reduced, and left ventricular ejection fraction (LVEF) was increased in the study group (*p* < 0.05). Compared to control group, six-minute walking test (6-MWT) distance was longer, and Minnesota Living with Heart Failure Questionnaire (MLHFQ) score and levels of inflammatory markers (IL-6, TNF-*α*, and hs-CRP) were lower in the study group (all *p* < 0.05). No significant difference was observed in the total incidence of adverse reactions between the two groups (*p* = 0.542).

**Conclusion:**

QSYQ combined with conventional Western medicine yielded a notable therapeutic effect on CHF, indicating that QSYQ may be an important adjuvant therapy for CHF patients.

## Introduction

Chronic heart failure (CHF) is a clinical syndrome characterized by structural and/or functional abnormalities of the heart, breathlessness, reduced exercise tolerance, and generalized edema (1, 2). The number of patients with heart failure is estimated to be around 64 million worldwide, and there are approximately 8.9 million cases in China among them, with the incidence is still on the rise (3, 4). Some modern medications have been used for the treatment of CHF in clinical practice, whereas the effect is not satisfactory and there are some adverse reactions (5–7). Therefore, there is an urgent need to explore more effective and safer complementary therapies for CHF patients.

Traditional Chinese medicine (TCM) has garnered increased attention in recent years, and has been recognized as an important component of complementary therapies for CHF (8). Qishen Yiqi dropping pills (QSYQ) is a TCM formula consisting of *Astragalus mongholicus*, *Salvia miltiorrhiza*, *Panax notoginseng*, and *Dalbergia odorifera* extracts (9). Salvianolic acid extracted from radix salviae miltiorrhiza can regulate inflammatory signaling pathways, and ginsenosides extracted from *Panax notoginseng* are found to activate endothelial nitric oxide synthase and promote vasodilation, thereby improving endothelial function (7). Evidence has shown that QSYQ promotes blood circulation, removes blood stasis, induces diuresis, and reduces swelling (10), thereby improving cardiac function, enhancing myocardial contractility, and increasing cardiac output (11). Bai et al. (12) have reported that QSYQ combined with Western medicine showed a lower risk of cardiac death, non-fatal myocardial infarction, and urgent revascularization than Western medicine alone in patients with acute coronary syndrome after percutaneous coronary intervention. A cohort study has reported that QSYQ combined with Western medicine improved ejection fraction classification and quality of life in patients with ischemic heart failure compared to Western medicine alone (13). However, evidence supporting the combined use of QSYQ and conventional Western medicine treatment in CHF patients needs to be further explored.

Therefore, our study aimed to investigate the effects and safety of combining QSYQ with conventional Western medicine on cardiac function and quality of life in CHF patients, which may offer valuable insights for CHF treatment strategies.

## Methods

### Study design

This was a single-center randomized controlled trial (RCT) performed from June 2022 to June 2024 in Shahe Hospital where patient recruitment, intervention, outcome assessment, and follow-up occurred. Statistical analysis was performed in Yan’an People’s Hospital. This study has been approved by the Institutional Ethics Committee of Shahe Hospital (Approval Reference: meeting date 2022-03-31), and prospectively registered on Chinese Clinical Trial Registry (Registration Number: ChiCTR2400084461) on May 16, 2024. All procedures were performed in accordance with the ethical standards of the institutional and/or national research committee and the 1964 Helsinki Declaration and its later amendments or comparable ethical standards. All participants have provided the written informed consent for this study, and randomly divided into control group (conventional Western medicine treatment) and study group (conventional Western medicine treatment + QSYQ) in a 1:1 ratio. Clinical Events Committee (CEC) or a Data and Safety Monitoring Board (DSMB) was not adopted in this study. Safety monitoring and outcome assessment were performed by the researcher throughout the trial.

### Participants

Inclusion criteria: (1) CHF patients with left ventricular ejection fraction (LVEF) ≤40%; (2) patients classified as grades II–IV according to the New York Heart Association (NYHA); (3) both patients and their family members voluntarily signed the informed consent form.

Exclusion criteria: (1) with mental disorders; (2) with acute heart diseases such as acute myocardial infarction; (3) with communication or hearing impairments; (4) with cerebrovascular diseases; (5) with chronic obstructive pulmonary disease; (6) patients who were allergic to QSYQ; (7) patients who had previously undergone cardiac intervention or bypass surgery; (8) with abnormal coagulation function; (9) patients who discontinued treatment midway or lost to follow-up for any reason.

CHF was determined by the Western medicine diagnostic criteria as outlined in the “Chinese Guidelines for the Diagnosis and Treatment of Heart Failure 2018.” The diagnosis was confirmed by evaluating the patient’s medical history, clinical symptoms and signs, and combining chest X-rays and echocardiograms. The TCM diagnostic criteria adhered to the “Guidelines for Clinical Research of New Chinese Medicines.”

### Sample size calculation

The sample size was estimated based on the reported efficacy rate in previous literature (14). The two-sided *α* level was 0.05 and *β* level was 0.20, thereby we calculated that each group needed 48 patients. Assuming a dropout rate of 20%, 60 patients were sufficient in each group, and a total of 120 patients were finally needed in this study.

### Randomization and blinding

Randomization was conducted by a computer, which generated a sequential number (001-120). The sequential number was placed in an opaque sealed envelope. According to the design of the random procedure, the researcher opened a random envelope upon the patient was enrolled and thereby obtained the sequential number of this patient and implemented corresponding allocation.

Participants and statistical analyst were blinded for the allocation of this study. Researcher and outcome assessor were not blinded because the researcher was responsible for both clinical treatment and outcome assessment. In addition, the characteristic appearance and smell of QSYQ made complete blinding impracticable. To reduce potential assessment bias, all outcome indicators were evaluated in strict accordance with standardized operating procedures.

### Treatments

The control group received guideline-directed conventional Western medicine treatment for heart failure, including angiotensin-converting enzyme inhibitor (ACEI)/angiotensin II receptor blocker (ARB), angiotensin receptor neprilysin inhibitor (ARNI), beta-blockers, mineralocorticoid receptor antagonists (MRAs), sodium-glucose cotransporter-2 (SGLT2) inhibitors, vasodilators, diuretics, and positive inotropic drugs. All patients were administered at least one class of the above medications. For ACEIs/ARBs, ARNI, beta-blockers, MRAs, and SGLT2 inhibitors, dosages were individually titrated to evidence-based target doses according to current clinical guidelines and patient tolerance.

The study group received the same conventional Western medical therapy as the control group, plus QSYQ (Tasly Holding Group Co., Ltd., National Approval No. Z20113048). QSYQ was administered orally at a dosage of 0.5 g per time, three times daily, and the total treatment duration in both groups was 6 months.

### Collection of serum samples

Before and 6 months following treatment, 5 mL of fasting peripheral venous blood was obtained from patients and placed in pre-cooled tubes. The tubes were left at 4 °C for 1 h to fully coagulate. Then, they were centrifuged at 4000 r/min for 10 min to separate the clear upper layer of serum.

### Measurement outcomes

The primary outcome was clinical efficacy, and the secondary outcomes were left ventricular end-systolic diameter (LVESD), left ventricular end-diastolic diameter (LVEDD), LVEF, six-minute walking test (6-MWT), quality of life, NT-proBNP, IL-6, TNF-*α*, hs-CRP, plasma viscosity, hematocrit, high shear rate whole blood viscosity and low shear rate whole blood viscosity, von Willebrand factor (vWF), endothelin-1 (ET-1), nitric oxide (NO), and adverse reactions.

Clinical efficacy of the two groups was compared after 6 months of treatment, and evaluated as marked effectiveness, effectiveness, and ineffectiveness. Marked effectiveness was defined that clinical symptoms such as shortness of breath and fatigue disappeared, and NYHA cardiac function improved by ≥2 grades or reached grade I. Effectiveness was defined that clinical symptoms improved, and NYHA cardiac function improved by 1 grade but not reached grade I. Ineffectiveness was defined that clinical symptoms and NYHA cardiac function grade did not meet the above standards. The total effectiveness rate = (number of marked effectiveness cases + number of effectiveness cases)/total number of cases × 100%.

LVESD, LVEDD, and LVEF were measured before and 6 months after treatment by the G4 xMatriz iU22 color ultrasound diagnostic instrument (Philips Company, Netherlands).

6-MWT was used to evaluate the exercise tolerance, and performed according to American Thoracic Society (ATS) standards (15). In a straight corridor approximately 30 meters long with a hard surface, patients were asked to walk as far as possible within 6 min. During the test, patients were not permitted to run or jog, but allowed to slow down, stop, or rest if necessary. The tests were administered and supervised by trained occupational therapists. After 6 min, the walking distance was recorded in meters and used for analysis.

The quality of life was assessed by Minnesota Living with Heart Failure Questionnaire (MLHFQ) (16), which contained 21 assessment items, and each item ranged from 0 to 5 points. The total score was 105 points, and higher score indicated the worse quality of life.

The serum levels of NT-proBNP, IL-6, TNF-*α*, and hs-CRP were detected using the enzyme-linked immunosorbent assay (ELISA, Nanjing Jiancheng Biotechnology Research Institute, China).

The plasma viscosity, hematocrit, high shear rate whole blood viscosity, and low shear rate whole blood viscosity were measured using the LG-R-80A hemorheometer (Shanghai Hanfei Medical Devices Company, China).

Vasoactive substances including vWF, ET-1 and NO were detected. vWF and ET-1 were determined by ELISA method (Nanjing Jiancheng Biotechnology Research Institute, China), and NO was determined by nitrate reduction method (Shanghai Saipeisen Biotechnology Company, China).

Adverse reactions included nausea and vomiting, rash, hypotension, gastrointestinal reaction, as well as dizziness and headache. The total incidence rate of adverse reactions was also compared between the two groups.

### Statistical analysis

Statistical analysis was performed utilizing SPSS 15.0 software. Categorical variables were presented as percentages or frequencies, whereas continuous variables were reported as means ± standard deviations. For categorical variables, the chi-square test was employed to measure differences. When comparing continuous variables with normal distributions between groups, the Student’s *t*-test was utilized. For continuous variables with abnormal distributions, the Wilcoxon rank-sum test was applied. A statistically significant difference was defined as *p* < 0.05.

## Results

### Selection and baseline characteristics of the included patients

**Figure 1:**
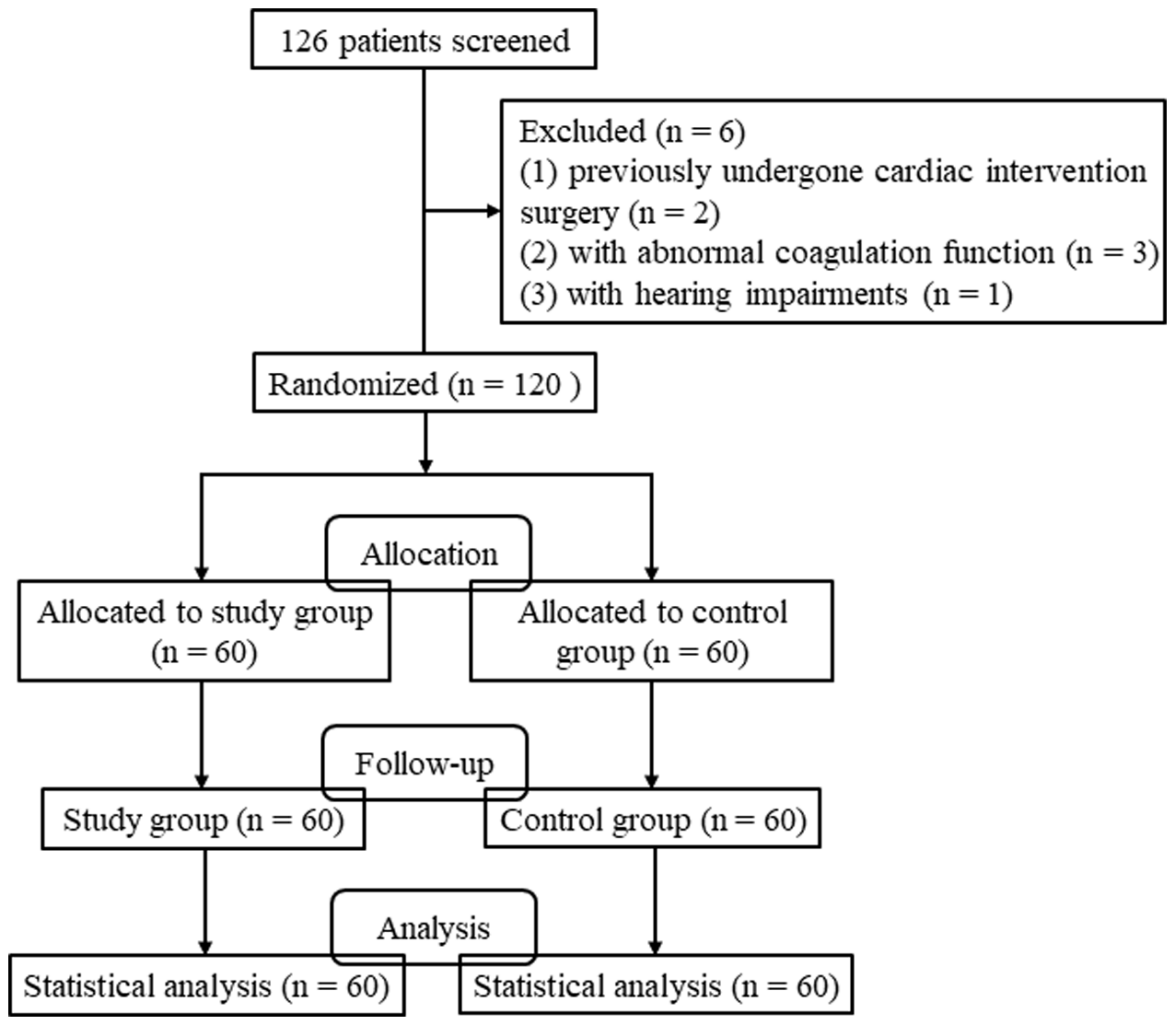
CONSORT flow diagram of this study.

In this study, 126 patients were screened. Of these, 2 patients previously undergone cardiac intervention surgery, 3 patients with abnormal coagulation function and 1 patient with hearing impairments were excluded. Finally, 120 patients were randomized and included for analysis, with 60 patients in the study group and 60 patients in the control group (Figure 1). Table 1 shows the baseline characteristics of the included patients. There was no significant difference in gender, age, BMI, previous medical history, oral medications, NYHA cardiac function classification, and primary etiology between the two groups (*p* > 0.05), suggesting that the two groups were comparable.

**Table 1:** Baseline characteristics between the two groups.

Characteristics	Control group (*n* = 60)	Study group (*n* = 60)	*χ*^2^/*t*	*p*
Gender			0.138	0.709
Male	35 (58.33)	37 (61.67)		
Female	25 (41.67)	23 (38.33)		
Age (years)	69.63 ± 14.49	70.32 ± 15.38	0.252	0.800
BMI (kg/m^2^)	22.97 ± 3.70	23.05 ± 4.03	0.113	0.910
History of coronary heart disease	35 (58.33)	37 (61.67)	0.138	0.709
History of hypertension	32 (53.33)	30 (50.00)	0.133	0.714
History of diabetes	21 (35.00)	22 (36.67)	0.036	0.849
History of smoking	18 (30.00)	20 (33.33)	0.154	0.694
History of drinking	50 (83.33)	48 (80.00)	0.222	0.637
Furosemide	48 (80.00)	46 (76.67)	0.196	0.657
Spirolactone	49 (81.67)	52 (86.67)	0.562	0.453
ACEI/ARB/ARNI	39 (65.00)	41 (68.33)	0.150	0.698
β-blocker	43 (71.67)	40 (66.67)	0.351	0.553
Digoxin	33 (55.00)	35 (58.33)	0.220	0.638
NYHA cardiac function classification			0.455	0.796
Grade II	11 (18.33)	13 (21.67)		
Grade III	31 (51.67)	32 (53.33)		
Grade IV	18 (30.00)	15 (25.00)		
Primary etiology			0.853	0.356
Ischemic	23 (38.33)	28 (46.67)		
Non-ischemic	37 (61.67)	32 (53.33)		

BMI, body mass index; ACEI, angiotensin-converting enzyme inhibitor; ARB, angiotensin II receptor blocker; ARNI, angiotensin receptor neprilysin inhibitor; NYHA, New York Heart Association.

### Clinical efficacy

Table 2 displays that there were 45% of patients with marked effectiveness and 46.67% of patients with effectiveness in the study group. Patients with marked effectiveness and effectiveness in control group were 33.33 and 36.67%, respectively. The study group had a higher total effective rate than the control group (91.67% vs. 70.00) (*p* = 0.002).

**Table 2:** Clinical efficacy between the two groups.

Groups	*N*	Marked effectiveness	Effectiveness	Ineffectiveness	Total effective rate
Control group	60	20 (33.33)	22 (36.67)	18 (30.00)	42 (70.00)
Study group	60	27 (45.00)	28 (46.67)	5 (8.33)	55 (91.67)
*χ* ^2^					9.090
*p*					0.002

### Cardiac function

**Figure 2:**
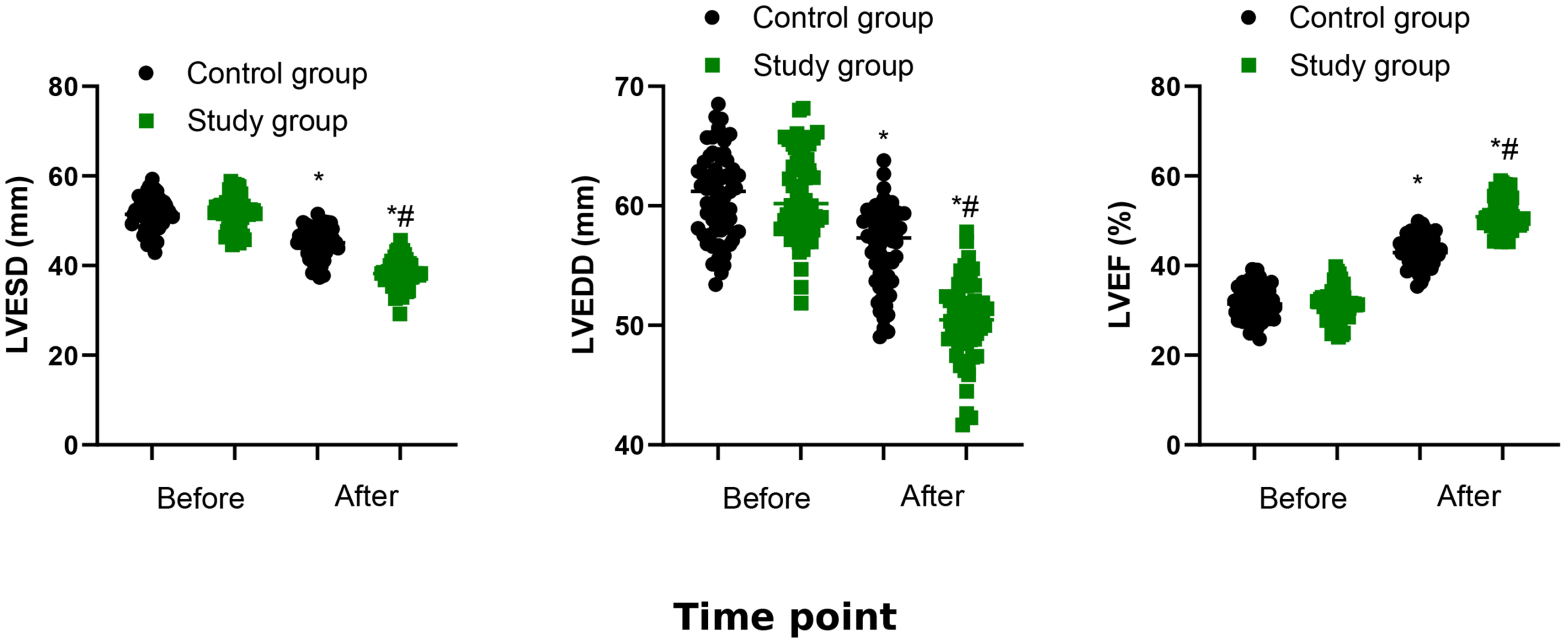
Cardiac function between the two groups. ^*^p < 0.05, vs. before treatment; ^#^*p* < 0.05, vs. control group.

Compared with before treatment, LVESD and LVEDD were declined, and LVEF was elevated after 6 months of treatment in both groups (*p* < 0.05). Compared to control group, the study group had lower LVESD and LVEDD as well as higher LVEF after the treatment (*p* < 0.05, Figure 2). The quantitative results were also shown in Table 3.

**Table 3:** Comparison of secondary outcomes.

Outcome	Before treatment	After treatment	*p* ^1^	*p* ^2^	*p* ^3^
LVESD, mean ± SD			<0.001	<0.001	<0.001
Control group	51.43 ± 3.61	45.07 ± 3.21			
Study group	51.79 ± 3.68	38.22 ± 3.17			
LVEDD, mean ± SD			<0.001	<0.001	<0.001
Control group	60.84 ± 3.59	56.50 ± 3.42			
Study group	60.91 ± 3.61	50.33 ± 3.31			
LVEF, mean ± SD			<0.001	<0.001	<0.001
Control group	31.52 ± 3.63	43.33 ± 3.26			
Study group	31.14 ± 3.48	51.85 ± 3.71			
6-MWT, mean ± SD			<0.001	<0.001	<0.001
Control group	82.54 ± 9.68	123.49 ± 10.38			
Study group	81.67 ± 9.31	163.58 ± 11.21			
MLHFQ score, median (IQR)			<0.001	<0.001	<0.001
Control group	66.41 (59.69, 70.46)	45.32 (40.07, 49.49)			
Study group	65.21 (61.27, 68.96)	34.12 (28.11, 37.85)			
NT-proBNP, mean ± SD			<0.001	<0.001	<0.001
Control group	433.68 ± 40.51	272.19 ± 15.14			
Study group	425.31 ± 43.01	231.62 ± 11.31			
hs-CRP, mean ± SD			<0.001	<0.001	<0.001
Control group	37.45 ± 8.62	15.71 ± 3.17			
Study group	36.67 ± 7.35	7.31 ± 1.53			
TNF-α, mean ± SD			<0.001	<0.001	<0.001
Control group	31.31 ± 3.42	24.47 ± 2.89			
Study group	31.60 ± 3.89	17.21 ± 2.54			
IL-6, mean ± SD			<0.001	<0.001	<0.001
Control group	20.08 ± 2.12	15.46 ± 1.79			
Study group	20.21 ± 2.29	10.12 ± 1.18			
Plasma viscosity, mean ± SD			<0.001	<0.001	<0.001
Control group	1.46 ± 0.10	1.29 ± 0.14			
Study group	1.44 ± 0.10	1.03 ± 0.14			
Hematocrit, mean ± SD			<0.001	<0.001	<0.001
Control group	48.53 ± 5.16	37.45 ± 1.08			
Study group	48.54 ± 5.18	30.09 ± 1.03			
High shear rate whole blood viscosity (IQR)			<0.001	<0.001	<0.001
Control group	5.73 (5.41, 5.91)	4.46 (4.32, 4.68)			
Study group	5.62 (5.14, 6.21)	3.01 (2.74, 3.32)			
Low shear rate whole blood viscosity, mean ± SD			<0.001	<0.001	<0.001
Control group	15.63 ± 1.18	10.22 ± 1.47			
Study group	15.27 ± 1.19	7.14 ± 0.80			
vWF, mean ± SD			<0.001	<0.001	<0.001
Control group	210.63 ± 40.21	144.30 ± 38.09			
Study group	211.46 ± 42.28	103.28 ± 24.42			
ET-1, mean ± SD			<0.001	<0.001	<0.001
Control group	83.27 ± 12.75	72.34 ± 14.11			
Study group	78.26 ± 10.08	52.33 ± 11.44			
NO, mean ± SD			<0.001	<0.001	<0.001
Control group	63.90 ± 9.27	82.37 ± 11.23			
Study group	62.71 ± 10.62	96.49 ± 12.75			

LVESD, left ventricular end-systolic diameter; LVEDD, left ventricular end-diastolic diameter; LVEF, left ventricular ejection fraction; 6-MWT, six-minute walking test; vWF, von Willebrand factor; ET-1, endothelin-1; NO, nitric oxide.

*p*^1^ for comparison in control group before and after treatment; *p*^2^ for comparison in study group before and after treatment; *p*^3^ for comparison between control group and study group after treatment.

### Exercise tolerance

**Figure 3:**
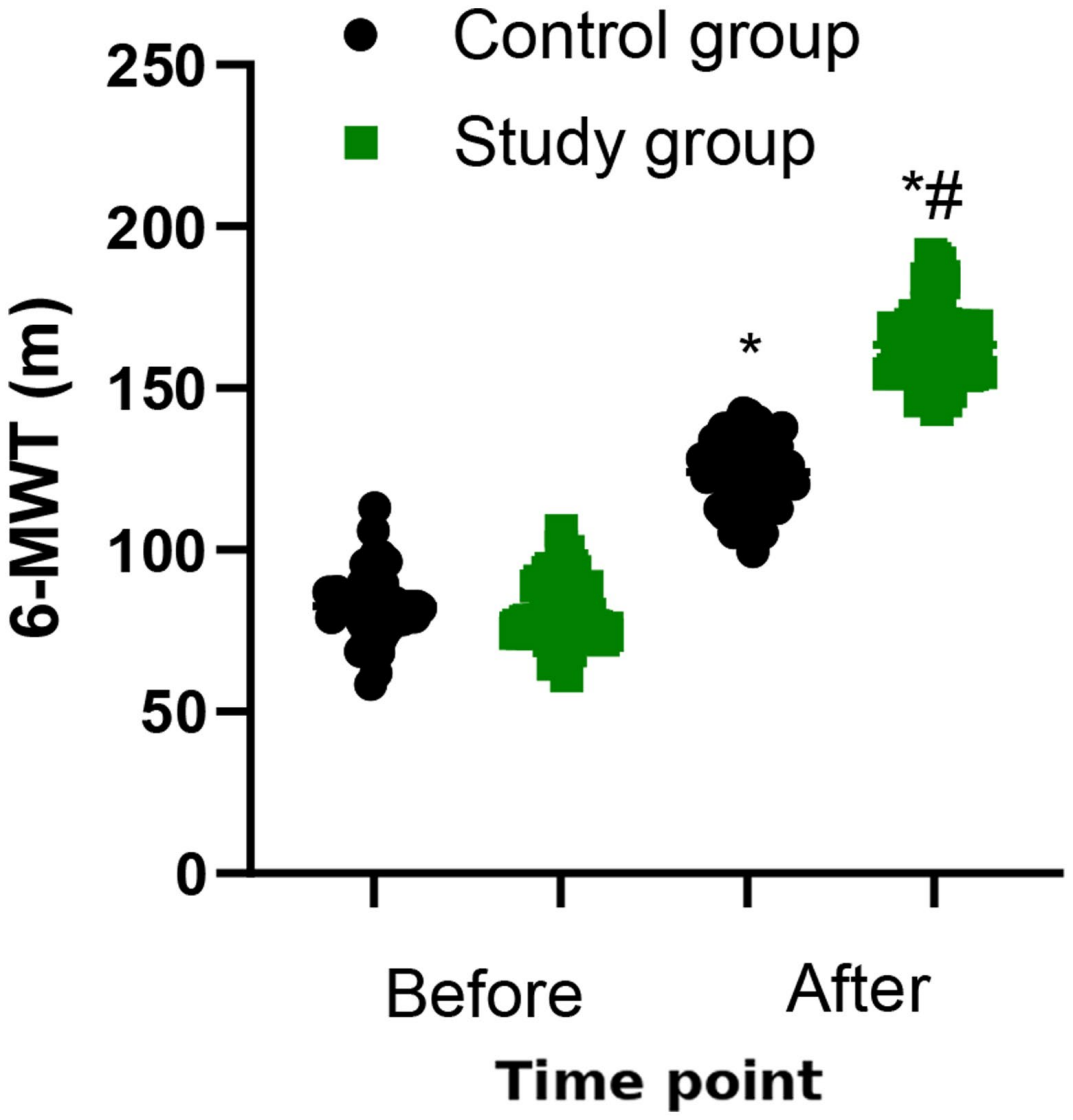
Exercise tolerance between the two groups. ^*^p < 0.05, vs. before treatment; ^#^*p* < 0.05, vs. control group.

The 6-MWT showed that exercise tolerance of the two groups were both elevated after 6 months of treatment (*p* < 0.05, Figure 3). Compared to control group, the study group displayed a better exercise tolerance (*p* < 0.001, Table 3).

### Quality of life

**Figure 4:**
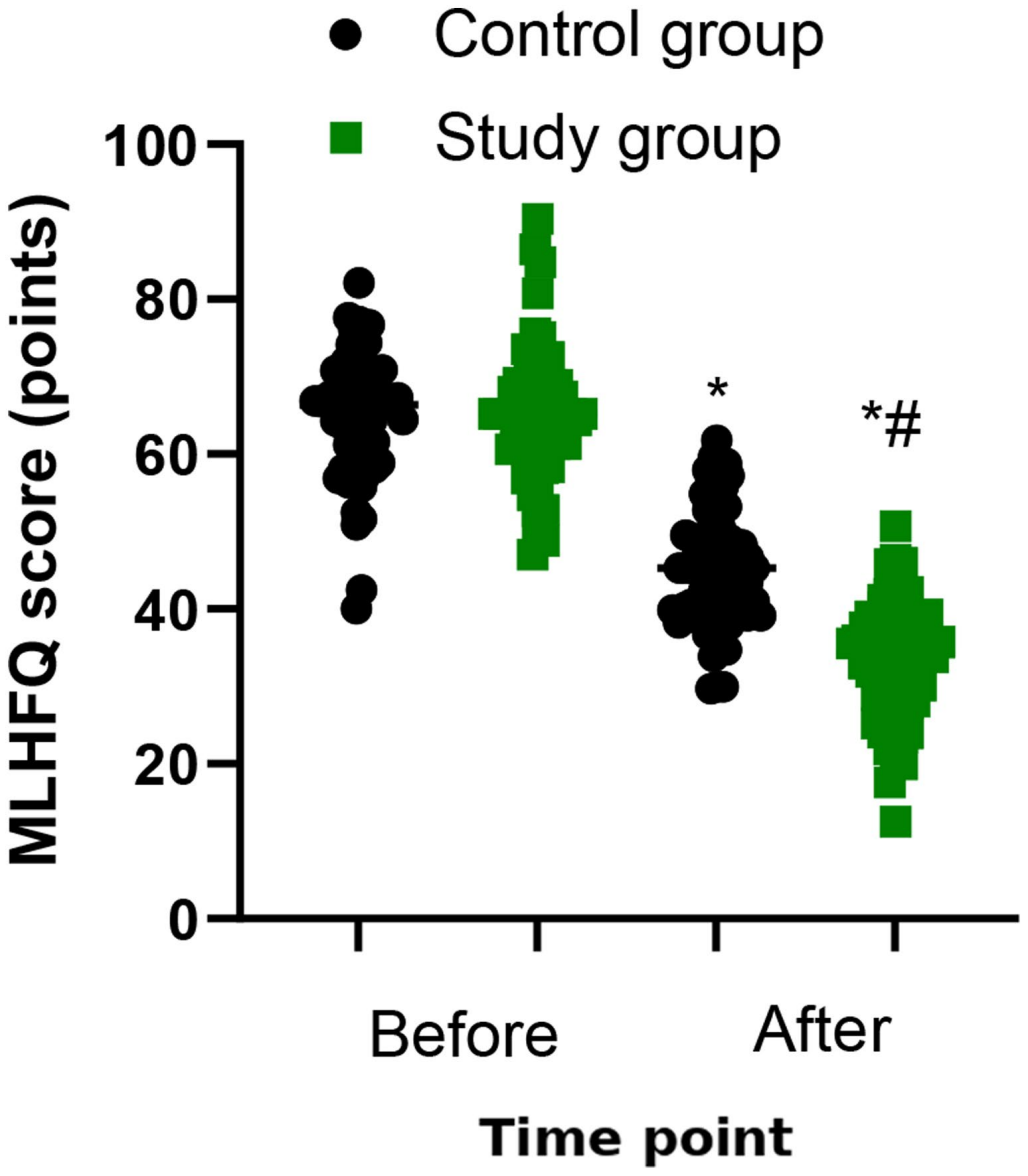
Quality of life between the two groups. ^*^p < 0.05, vs. before treatment; ^#^*p* < 0.05, vs. control group.

The MLHFQ score of the both groups were significantly reduced after 6 months of treatment (*p* < 0.05, Figure 4). Compared to control group, the study group had a lower MLHFQ score after the treatment (34.12 vs. 45.32) (*p* < 0.001, Table 3).

### Serological indicators

**Figure 5:**
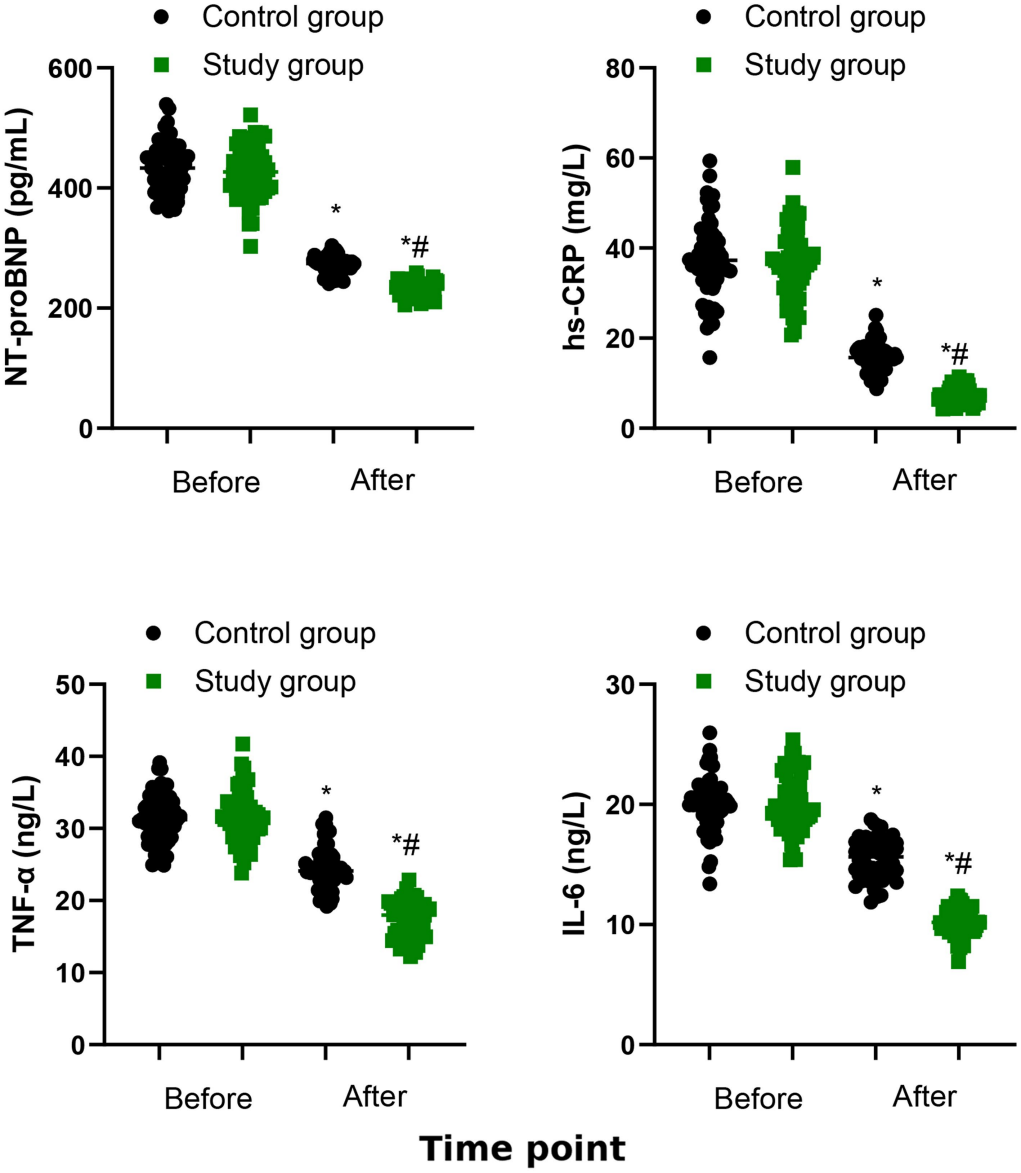
Serological indicators between the two groups. ^*^p < 0.05, vs. before treatment; ^#^*p* < 0.05, vs. control group.

Figure 5 shows that the serum levels of NT-proBNP, hs-CRP, TNF-*α*, and IL-6 of the two groups were both declined after 6 months of treatment (*p* < 0.05). The study group had lower serum levels of NT-proBNP, hs-CRP, TNF-α, and IL-6 than the control group (*p* < 0.001, Table 3).

### Hemodynamic indexes

**Figure 6:**
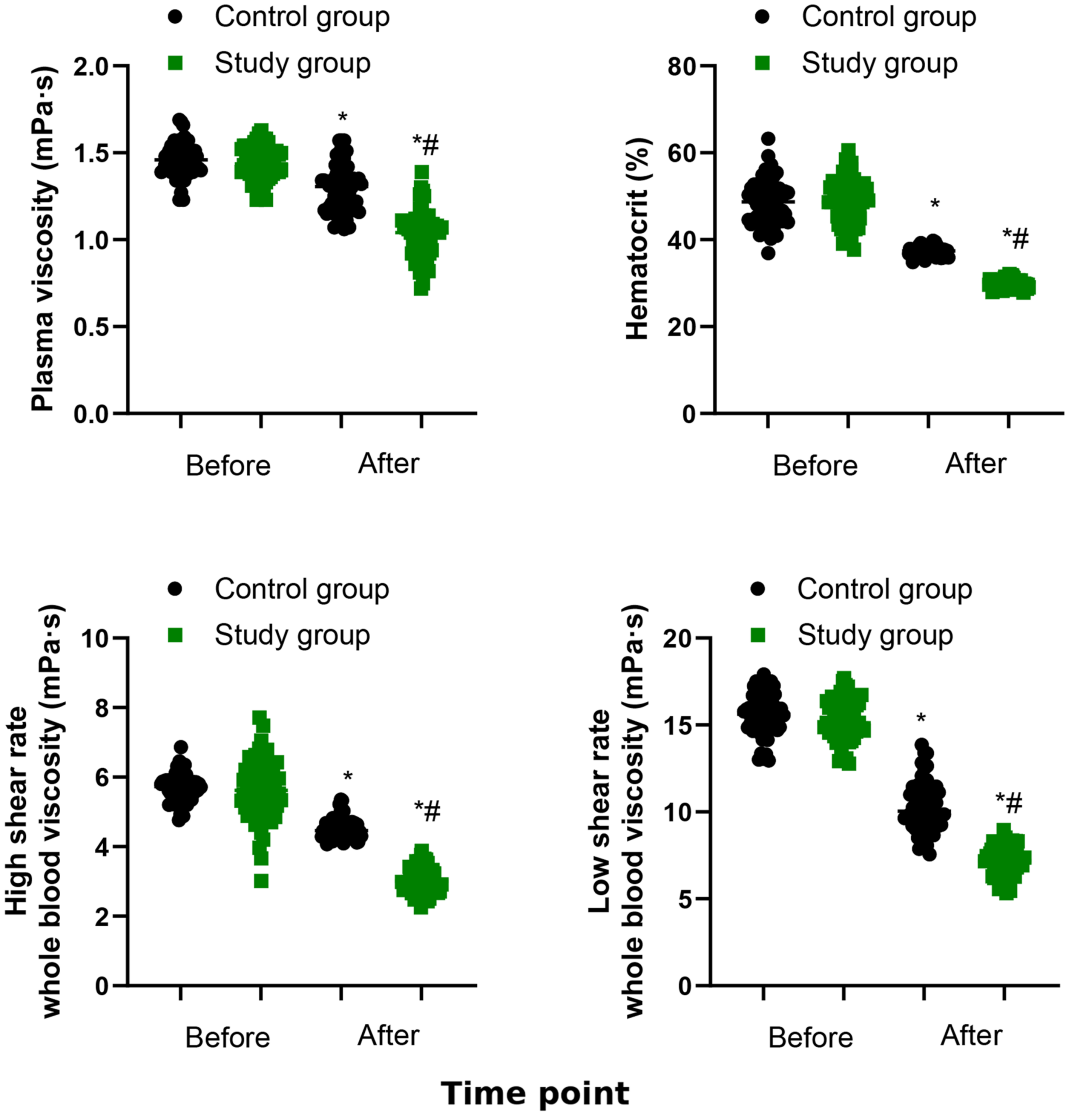
Hemodynamic indexes between the two groups. ^*^p < 0.05, vs. before treatment; ^#^*p* < 0.05, vs. control group.

Figure 6 demonstrates that the plasma viscosity, hematocrit, high shear rate whole blood viscosity and low shear rate whole blood viscosity of the both groups were significantly declined after 6 months of treatment (*p* < 0.05). The study group had lower plasma viscosity, hematocrit, high shear rate whole blood viscosity and low shear rate whole blood viscosity than the control group (*p* < 0.001, Table 3).

### Vasoactive substances

**Figure 7:**
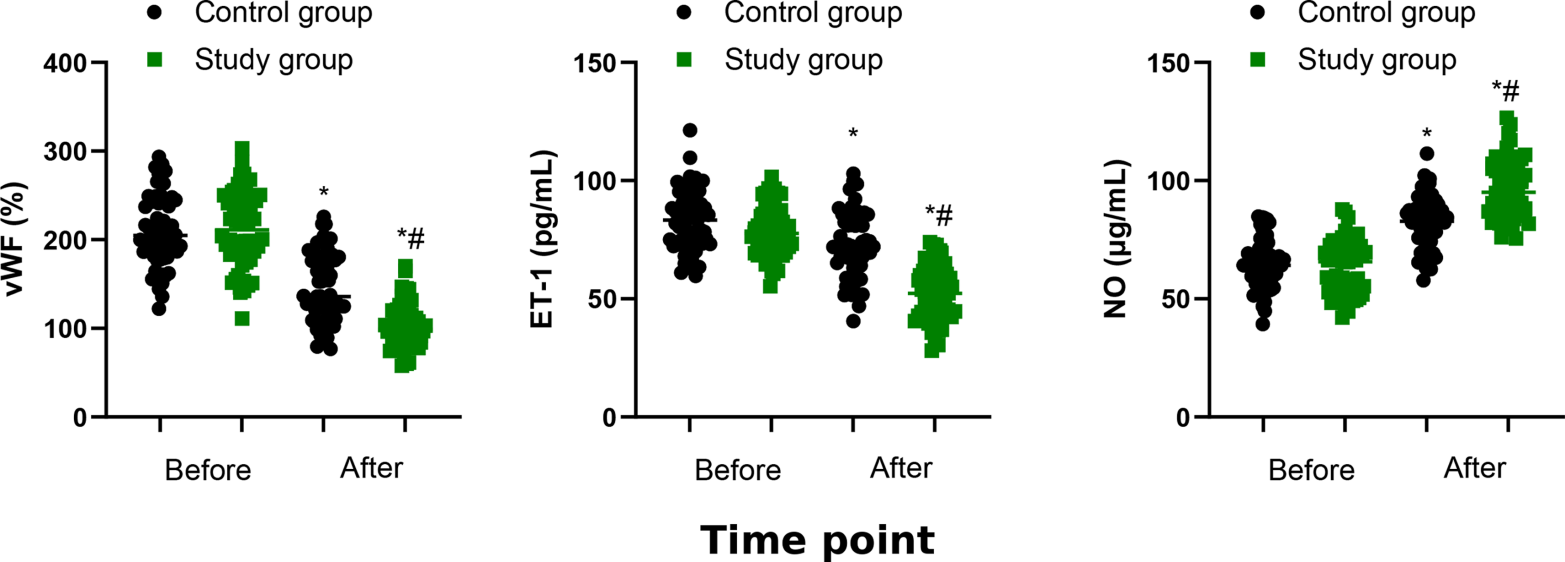
Vasoactive substances between the two groups. ^*^p < 0.05, vs. before treatment; ^#^*p* < 0.05, vs. control group.

The levels of vWF and ET-1 were reduced and the level of NO was increased in the two groups after 6 months of treatment (*p* < 0.05, Figure 7). The study group had lower serum levels of vWF and ET-1 as well as higher serum level of NO than the control group (*p* < 0.001, Table 3).

### Occurrence of adverse reactions

Table 4 displays that there was no significant difference in the total incidence rate of adverse reactions between the two groups after 6 months of treatment (*p* = 0.542).

**Table 4:** Occurrence of adverse reactions between the two groups.

Groups	*N*	Nausea and vomiting	Rash	Hypotension	Gastrointestinal reaction	Dizziness and headache	Total incidence rate
Control group	60	2 (3.33)	1 (1.66)	1 (1.66)	2 (3.33)	1 (1.66)	7 (11.64)
Study group	60	1 (1.66)	1 (1.66)	1 (1.66)	1 (1.66)	1 (1.66)	5 (8.30)
*χ* ^2^							0.370
*p*							0.542

## Discussion

CHF is the result of various cardiac diseases developing to the end stage and the effect of Western medicine alone is not satisfactory (7). The combined use of QSYQ and Western medicine has been reported to more effective than Western medicine alone in some heart diseases, including acute coronary syndrome and ischemic heart failure (12, 13). In this study, we explored the effect and safety of QSYQ combined with Western medicine in CHF patients, and results showed that the study group exhibited better clinical efficacy, lower LVESD and LVEDD as well as higher LVEF, longer 6-MWT, lower MLHFQ score, lower NT-proBNP, hs-CRP, TNF-*α* as well as IL-6 levels, lower plasma viscosity, hematocrit, high shear rate whole blood viscosity as well as low shear rate whole blood viscosity, lower vWF and ET-1 levels as well as higher NO levels than the control group.

Evidence has demonstrated that QSYQ can improve the overall function of left ventricular and myocardial structure and function abnormalities caused by ischemia–reperfusion injury (7). It was also reported to inhibit the increase of LVEDD, produce anti-ventricular remodeling effects, and protect extracellular matrix and cardiomyocytes, thereby effectively improving patients’ cardiac function (7). Ejection fraction has been an important indicator to evaluate the cardiac function of patients with heart failure (13). Chen et al. (17) have reported that QSYQ combined with Western medicine improved LVEF, LVEDD, LVESD, and 6-MWT in CHF patients. In addition, some studies have shown that QSYQ used in combination with standard heart failure treatment have significant advantages in improving clinical symptoms, enhancing exercise tolerance, and promoting quality of life in CHF patients (11, 18). Consistently, our results showed better clinical efficacy, higher LVEF, longer 6-MWT, and lower LVESD, LVEDD and MLHFQ score in study group, indicating that the combination of QSYQ and Western medicine may be an effective treatment for CHF patients by enhancing cardiac function, boosting exercise endurance, and promoting patients’ quality of life.

NT-proBNP is an important marker for heart failure, which is secreted by ventricular muscle cells in response to increased pressure load and reflect changes in ventricular wall tension (19). The inflammatory response plays a pivotal role in the progression of CHF (20). Wu et al. (21) have reported that inhibiting inflammatory responses and decreasing levels of inflammatory markers (hs-CRP, TNF-*α*, IL-6) are of great importance to prevent and manage CHF. Our study showed that the study group exhibited reduced levels of NT-proBNP, IL-6, TNF-α, and hs-CRP, suggesting that QSYQ combined with conventional Western medicine may suppress the inflammatory response in CHF patients. This was consistent with Luo’s et al. (22) findings that QSYQ decreased levels of inflammatory markers, such as IL-6 and TNF-α, in isoproterenol-induced cardiomyocytes.

Impaired blood circulation in CHF patients could elevate plasma osmotic pressure, leading to cellular dehydration. Prolonged tissue hypoxia may stimulate the liver to synthesize fibrinogen, resulting in hypercoagulability and altered blood flow velocity (23). Our research demonstrated lower plasma viscosity, hematocrit, high shear rate whole blood viscosity and low shear rate whole blood viscosity in study group. These findings implied that the combination of QSYQ and conventional Western medicine may improve hemorheology in CHF patients. This might be achieved through a synergistic effect that enhances ischemic myocardial energy metabolism, promotes vascular smooth muscle cell proliferation, inhibits platelet activation, improves red blood cell deformability, and optimizes blood rheology.

Vasoactive substances are involved in the pathogenesis of CHF. vWF and ET-1 could promote vascular contraction and thrombosis, which may result in vascular endothelial damage and induce myocardial remodeling (24). NO could inhibit platelet aggregation, and its reduction may lead to vascular endothelial dysfunction and CHF progression (25). Our study showed lower vWF and ET-1 levels and higher NO level in study group, indicating that QSYQ combined with conventional Western medicine may protect vascular endothelial function in CHF patients. This protection may be attributed to reduced myocardial oxygen consumption, alleviated inflammatory responses, inhibited thrombosis, delayed myocardial fibrosis, and improved microcirculation.

The present findings suggest that combination therapy with QSYQ and conventional Western medicine yields significant clinical benefits in CHF patients. However, some limitations of this study should be noticed. First, this is a single-center study with a relatively small sample size, which may limit the generalizability of the present findings. Second, the follow-up duration is relatively short. Future studies with bigger sample size are needed to further explore the long-term effect and safety of QSYQ combined with conventional Western medicine in CHF patients. Third, a blank control group is not included in this study. This study aims to investigate the efficacy of QSYQ as an adjuvant treatment, and conventional Western medicines are used in the control group, which may limit the interpretation of the independent therapeutic effect of QSYQ. Fourth, independent data monitoring is not performed during the trial; safety monitoring and outcome assessment are conducted by the researcher, who is not blinded due to the nature of the intervention and clinical practice requirements, which may introduce potential observer bias. However, standardized assessment procedures are applied to minimize this influence. Fifth, levels of inflammatory markers including IL 6, TNF *α*, and hs-CRP are significantly reduced and cardiac function is improved in the study group. However, the potential association between decreased inflammatory levels and the improved cardiac function is not explored in the present study. Further studies are needed to investigate the association between inflammatory markers and cardiac function parameters in CHF patients.

## Conclusion

The combination of QSYQ and conventional Western medicine exerts a remarkable therapeutic effect on CHF. It improves cardiac function, reduces inflammation, enhances hemorheology, boosts exercise endurance, promotes quality of life, protects vascular endothelial function, and demonstrates a certain degree of safety. These results indicate that QSYQ may serve as a promising and safe adjuvant intervention for optimizing clinical outcomes in CHF.

## Data Availability

The original contributions presented in the study are included in the article/supplementary material, further inquiries can be directed to the corresponding author.
